# New Insights in the Fight against HIV

**DOI:** 10.3390/cells10123549

**Published:** 2021-12-16

**Authors:** Daria Trabattoni, Mara Biasin

**Affiliations:** Department of Biomedical and Clinical Sciencies L. Sacco, University of Milan, 20157 Milan, Italy; daria.trabattoni@unimi.it

Effective antiviral immune responses rely on the host’s genetic background and its interaction with the surrounding environment. Chronic viral infections, including HIV, result in significant and often irreversible changes in the function of the immune system, among other organs, thus progressively affecting the course of disease if left unchecked. In addition to depleting one of the main components of our immune arsenal, namely CD4 T cells, the establishment of a latent infection characteristic of HIV, i.e., virus reservoir, makes life-long pharmacological treatment the only therapeutic option available to date.

A number of immunological and genetic correlates of susceptibility to infection have been identified over the years, either restricting HIV entry and/or its replication by directly interacting with the virus components or modulating mediators of the host antiviral/inflammatory response. Among the virus restriction factors, type I IFNs and the related interferon stimulated genes (ISGs) represent the first line of defense that virtually any cell can deploy to fight an infection. Such a response is activated when pathogen-associated molecular patterns (PAMPs), such as the virus genome, are recognized by cellular pattern recognition receptors (PRR), including Toll-like receptors (TLRs). 

In this Special Issue, Marziali and colleagues give a comprehensive overview of the mechanisms underlying the anti-HIV action of some key members of ISGs, namely, interferon-induced transmembrane proteins (IFITMs) [1], while Rojas et al. offer an interesting perspective on the potential role of type I IFNs and ISGs in the establishment of a latent infection in macrophages [2]. Of course, the same innate mechanisms may play a crucial role in the control of opportunistic and co-infection, such as that from cytomegalovirus (CMV), a ubiquitous herpesvirus among adults that often undergoes reactivation in immunocompromised individuals. Jabłońska and coworkers identify TLR9 polymorphisms as a novel correlate of the type I IFN response and CMV viremia in HIV co-infected subjects [3]. 

In addition to the host factors directly targeting the virus, the overall immunological milieu is of crucial importance in affecting local virus replication and pathogenesis, with a pro-inflammatory environment that is usually regarded as favorable to the virus. The group of Serrano-Rísquez shows how genetic variants in CD46, which protects the host from potentially deleterious effects of complement activation, are associated with resistance to HIV-1 infection [4]. However, a fine balance between pro- and anti-inflammatory stimuli is necessary to maintain immunological homeostasis at tissue sites of virus replication, as demonstrated by Boby and coworkers [5]. In a non-human primate model of HIV infection, the authors identified a dysregulated expression of the immunosuppressive molecule transforming growth factor (TGF) β, along with its cellular source, as a correlate of HIV pathogenesis in the intestine, one of the major sites supporting HIV replication and harboring latently infected cells. In Vanetti’s manuscript, HIV infection was leveraged as a platform to study host–pathogen interactions in the context of COVID-19, identifying increased levels of the immunosuppressive cytokine IL-10 driven by co-infection as a protective factor against SARS-CoV-2 in vivo as well as in vitro [6].

As an alternative to lifelong pharmacological therapy to keep patients in a status of virologic suppression, novel interventions to purge the HIV reservoir have been pursued in recent years with scarce success due to our poor understanding of the composition and mechanisms regulating latent infection. A research article by Wright et al. reports on the optimization and validation of novel analytical tools to characterize integrated HIV sequences with a single genome near full-length amplicon resolution [7]. Castelli and coauthors discuss the clinical impact of an effective class of anti-cancer drugs, i.e., immune checkpoint inhibitors, in HIV-infected subjects, also as a potential virus reservoir eradication strategy [8]. As cancer is a common comorbidity in immunocompromised individuals, the effect of drug–drug interaction between antiretrovirals and chemotherapeutics on liver toxicity is discussed by Bressan et al. [9]. Finally, although the feasibility of achieving protective immunity against HIV via vaccination is yet to be demonstrated, research by Jordan-Paiz and colleagues sheds light on the effect of synonymous codon pair recoding, an efficient strategy for virus attenuation of the HIV-1 env gene, on virus replication [10]. 

Although the scientific field of HIV infection is very open and there is still much to be done, this Special Issue provides new insights on the current molecular and cellular basis involved in the fight against this virus, both to understand the functional immune mechanisms and also for a potential translation into clinical practice.

## Data Availability

Not Applicable.
